# Post-Neoadjuvant Treatment Strategies in Breast Cancer

**DOI:** 10.3390/cancers14051246

**Published:** 2022-02-28

**Authors:** Christiane Matuschek, Danny Jazmati, Edwin Bölke, Bálint Tamaskovics, Stefanie Corradini, Wilfried Budach, David Krug, Svjetlana Mohrmann, Eugen Ruckhäberle, Tanja Fehm, Carolin Nestle Krämling, Markus Dommach, Jan Haussmann

**Affiliations:** 1 Department of Radiation Oncology, University Hospital Dusseldorf, Medical Faculty, Heinrich-Heine-University Dusseldorf, Moorenstr. 5, 40225 Dusseldorf, Germany; matuschek@med.uni-duesseldorf.de (C.M.); danny.jazmati@med.uni-duesseldorf.de (D.J.); balint.tamaskovics@med.uni-duesseldorf.de (B.T.); wilfried.budach@med.uni-duesseldorf.de (W.B.); jan.haussmann@med.uni-duesseldorf.de (J.H.); 2 Department of Radiation Oncology, LMU University of Munich, 81388 Munich, Germany; stefanie.corradini@med.uni-muenchen.de; 3 Department of Radiation Oncology, University Medical Center Schleswig-Holstein, 24105 Kiel, Germany; david.krug@uksh.de; 4 Department of Gynecology & Obstetrics, Heinrich-Heine University, 40225 Dusseldorf, Germany; mohrmann@med.uni-duesseldorf.de (S.M.); eugen.ruckhaeberle@med.uni-duesseldorf.de (E.R.); tanja.fehm@med.uni-duesseldorf.de (T.F.); 5 Department of Senology: EVK Dusseldorf, 40217 Dusseldorf, Germany; dr.nestle-kraemling@t-online.de; 6 Department of Internal Medicine, Sana Krankenhaus, 40625 Dusseldorf, Germany; markus.dommach@sana.de

**Keywords:** breast cancer, post-neoadjuvant, individualized treatment

## Abstract

**Simple Summary:**

In the treatment of patients with breast cancer, post-neoadjuvant approaches represent an attractive opportunity to improve patient outcomes by stratifying adjuvant treatment according to tumor response. Thus, these concepts represent a step towards our vision of individualized adaptive tumor treatment. Although apparently in its early stages, increasing evidence indicates an important change to our historical treatment strategies.

**Abstract:**

Neoadjuvant chemotherapy enables close monitoring of tumor response in patients with breast cancer. Being able to assess tumor response during treatment provides an opportunity to evaluate new therapeutic strategies. Thus, for triple-negative breast tumors, it was demonstrated that additional immunotherapy could improve prognosis compared with chemotherapy alone. Furthermore, adjuvant therapy can be escalated or de-escalated correspondingly. The CREATE-X trial randomly assigned HER2-negative patients with residual tumor after neoadjuvant therapy to either observation or capecitabine. In HER2-negative patients with positive BRCA testing, the OlympiA study randomly assigned patients to either observation or olaparib. HER2-positive patients without pathologic remission were randomly assigned to trastuzumab or trastuzumab–emtansine within the KATHERINE study. These studies were all able to show an improvement in oncologic outcome associated with the escalation of therapy in patients presenting with residual tumor after neoadjuvant treatment. On the other hand, this individualization of therapy may also offer the possibility to de-escalate treatment, and thereby reduce morbidity. Among WSG-ADAPT HER2+/HR-, HER2-positive patients achieved comparable results without chemotherapy after complete remission following neoadjuvant treatment. In summary, the concept of post-neoadjuvant therapy constitutes a great opportunity for individualized cancer treatment, potentially improving outcome. In this review, the most important trials of post-neoadjuvant therapy are compiled and discussed.

## 1. Introduction

Historically, neoadjuvant systemic therapy was generally reserved for unresectable or inflammatory breast cancer. In a randomized comparison of neoadjuvant and adjuvant systemic therapy using doxorubicin and cyclophosphamide, the analysis revealed no difference in freedom of disease and survival [1]. However, the study showed a significant reduction in tumor burden in the breast, allowing patients to avoid mastectomy and opt for a breast-conserving surgical approach instead. Moreover, the trial also showed a strong correlation between tumor response to the primary systemic therapy and oncologic outcomes. This allowed trialists to dichotomize the patient population into two strata and apply adjuvant treatment (post-neoadjuvant) according to the pathological response to the neoadjuvant systemic treatment. This strategy permits studying new systemic therapies or new combinations of treatment modalities. More important, however, is that we can now use this refined or individualized prognosis assessment to either escalate postoperative therapy (if the risk of recurrence remains high) or to de-escalate it (if the prognosis is favorable). Since such a risk-adapted adjuvant treatment is only possible through a neoadjuvant application of systemic therapy, this is referred to as post-neoadjuvant therapy.

Three different strategies for the implementation of new therapeutics were followed in the currently published randomized trials. First, the addition of new substances in the neoadjuvant phase, which was continued in the adjuvant setting [2,3]. Second, testing new regimes in trials in the adjuvant setting, which included patients that were treated with systemic therapy either before surgery or solely after surgery [4]. Third, currently, the most popular approach is to intensify or de-intensify treatment after a standard neoadjuvant therapy and then test new drugs according to response [5,6,7].

This review summarizes the available scientific data and discusses their impact on the management of breast cancer. The most important trials are shown in Table 1 and the ongoing trials in Table 2.

## 2. Post-Neoadjuvant Chemotherapy/Immunotherapy

Taking into account the response to systemic therapy administered preoperatively allows us to estimate the prognosis of the individual patient much more precisely than was previously possible with initial diagnosis on the basis of tumor stage and tumor biology. Therefore, if chemotherapy and/or targeted therapy are indicated for primary, curatively treatable breast cancer, this should be administered neoadjuvantly, i.e., as the first treatment before surgery [13,14,15]. In addition to a higher rate of breast-conserving therapies, this approach also allows in vivo sensitivity testing. This means that the response of the breast tumor to the neoadjuvant systemic therapy can be used to estimate its effect on the destruction or control of micrometastases and, throughout this, the prognostic effect, the actual goal of any systemic therapy in the treatment of primary breast cancer.

One randomized phase III study demonstrated the advantage of additional capecitabine after neoadjuvant chemotherapy [6,16]. In this study, 910 patients with HER2-negative disease and residual disease following neoadjuvant chemotherapy were treated with or without capecitabine as post-neoadjuvant treatment. Both disease-free survival and overall survival in the capecitabine group were significantly (*p* = 0.01) superior to that of the control group at 5 years, respectively (74.1% vs. 67.6% and 89.2% vs. 83.6%). In subgroup analysis, it could be demonstrated that this benefit was restricted to patients with triple-negative disease. However, substantially more high-grade complications were reported for the capecitabine group, particularly hand–foot syndrome, hematologic side effects and diarrhea. All complications were nonfatal and only transient. A subsequent phase III trial by the ECOG-ACRIN group failed to demonstrate the superiority of post-neoadjuvant treatment with carboplatin or cisplatin over capecitabine in triple-negative breast cancer patients with residual disease after neoadjuvant chemotherapy [10].

Several trials tested the addition of immune checkpoint blockers in the neoadjuvant and adjuvant treatment of triple-negative breast cancer [2,3,11,12]. The German GeparNuevo study was able to demonstrate a non-significant improvement in the pCR rate, and even a significantly improved survival rate, in primary triple-negative breast cancer patients with the addition of durvalumab to neoadjuvant chemotherapy [11]. These results have been confirmed within the phase III Keynote 522 trial for high-risk, early-stage and triple-negative breast cancers treated with neoadjuvant chemotherapy, with or without pembrolizumab [3].

At the same time, post-neoadjuvant therapy offers the opportunity to identify chemo-resistant subgroups and broadens the scope for individualized treatment. Using a combination of everolimus and cisplatin, Anand et al. presented a therapeutic approach to target crucial mechanisms of chemotherapy resistance during the post-neoadjuvant setting of 20 patients with triple-negative breast carcinoma [17].

## 3. Residual Disease following Neoadjuvant Therapy

Post-neoadjuvant strategies in breast cancer are a smart set-up for adjuvant clinical trials, presenting the chance to investigate new drugs or combinations in high-risk patients who did not accomplish pathologic complete response after primary treatment.

The first comparative study that selected a high subgroup (triple-negative breast cancer patients without a pCR after surgery) in the post-neoadjuvant setting was the CREATE-X study, showing that this individualization of postoperative therapy can improve survival outcomes [6,18]. The Japanese and Korean randomized clinical trial (CREATE-X) of capecitabine versus no additional therapy has been conducted in women with early-stage breast cancer who received standard neoadjuvant chemotherapy and had residual invasive breast cancer at the time of surgery. The results from the CREATE-X trial demonstrate that capecitabine has a statistically significant survival advantage compared with no additional therapy [6].

Unfortunately, compared with patients with pathologic complete response, the risk of recurrence is significantly higher in patients with residual disease following neoadjuvant chemotherapy, indicating a potential resistance to systemic treatment. There is currently only some evidence for these patients, except for HER2-positive and triple-negative patients. This is compounded as, until today, only some studies have prospectively investigated this concept.

The landmark randomized KATHERINE trial involved 1486 patients with HER2 positive early breast cancer, who were found to have residual invasive disease in the breast or axilla at surgery following neoadjuvant therapy with taxanes (with or without anthracycline) and trastuzumab. In this trial, patients were randomly assigned to adjuvant trastuzumab emtansine (T-DM1) or continuation of trastuzumab for 14 cycles. Von Minckwitz et al. reported that the risk of recurrence of invasive breast cancer or death was significantly reduced with adjuvant T-DM1 as compared with trastuzumab alone [5]. Currently, the DESTINY-Breast05 trial (NCT04622319) is enrolling patients and aims to compare the efficacy of T-DM1 with Trastuzumab Deruxtecan in the post-neoadjuvant setting.

In this context, the findings after neoadjuvant therapy can be used as an in vivo sensitivity test, and further tailored treatment can improve the outcome in high-risk patients. To date, for triple-negative, and for HER2-positive patients without pathologic complete response, there exists clear evidence for a prognostic beneficial post-neoadjuvant treatment.

Several trials are currently addressing this post-neoadjuvant setting. Therefore, the treatment of patients in the post-neoadjuvant setting remains a clinical challenge, with limited data supporting the use of additional post-neoadjuvant chemotherapy or other additional treatment options including, e.g., targeted therapy or immunotherapy.

## 4. PARP Inhibitors

The specific high-risk subgroup of women carrying the BRCA mutation that were diagnosed with high-risk breast cancer was addressed in the multi-center international phase III OlympiA-trial. The authors demonstrated that in patients with HER2-negative BRCA-positive tumor after local therapy and neoadjuvant or adjuvant chemotherapy, adjuvant therapy with olaparib was superior to placebo in terms of reducing mortality (*p* = 0.02), disease- (*p* < 0.001), and distant free survival (*p* < 0.001) [4]. One criterion for inclusion in the study was residual tumor after systemic therapy in patients receiving neoadjuvant chemotherapy. This applied to approximately half of the 1836 patients. A subgroup analysis of these patients would be highly desirable, as those patients might have benefitted most from treatment escalation.

## 5. ER-Positive/HER2-Negative Disease and CDK4/6 Inhibitors

The positive prognostic value of a tumor response to neoadjuvant therapy including chemotherapy and anti-HER2 antibody therapy, but also for endocrine therapy, has been proven for all breast cancer subtypes. This is why the concept of post-neoadjuvant therapy has been applied to hormone receptor-positive tumors.

In the PENELOPE-B trial, 1250 women with hormone receptor-positive, HER2-negative primary breast cancer without a pathological complete response after taxane-containing neoadjuvant chemotherapy, and at high risk of relapse, were randomized to receive 13 cycles of palbociclib 125 mg once daily or placebo. The median age was 49.0 years, and the majority were ypN+ with a Ki-67 ≤ 15%. After a median follow-up of 42.8 months, palbociclib did not improve invasive disease-free survival versus placebo and resulted in a stratified hazard ratio of 0.93 (*p* = 0.525). Additionally, they did not find any differences among the subgroups [7]. Different results have been reported by the monarchE trial investigating the role of abemaciclib in HR-positive high-risk patients after adjuvant or neoadjuvant therapy (37% neoadjuvant chemotherapy). Both subgroups demonstrated a significant IDFS benefit by adding the CDK 4/6 inhibitor abemaciclib to endocrine treatment [19]. Currently, due to the controversial results, routine use of adjuvant CDK4/6 inhibitors is not universally recommended.

## 6. De-Escalation Approaches

While the above-mentioned trials and approaches mostly represent treatment escalation in patients with unfavorable response to neoadjuvant therapy, there is also the potential to de-escalate treatment in patients with a favorable response. For systemic therapy, this has been addressed in patients with HER2-positive disease. The PREDIX HER2-trial compared docetaxel, trastuzumab and pertuzumab to T-DM1 and found similar rates of pathologic complete response and event-free survival in both arms [20,21]. Results on survival outcomes are pending. Furthermore, the included treatment response approach by PET/CT needs further evaluation.

The KRISTINE trial randomized patients to neoadjuvant T-DM1 and pertuzumab vs. docetaxel, carboplatin, trastuzumab and pertuzumab [22]. Rates of pathologic complete response were significantly lower with de-escalated neoadjuvant treatment, and increased rates of locoregional progression before surgery led to inferior rates of invasive disease-free survival. However, treatment tolerability and patient-reported outcomes favored the T-DM1 plus pertuzumab arm. Similarly, chemotherapy-free treatment with trastuzumab and pertuzumab resulted in inferior rates of pathologic complete response when compared with the same regimen with additional paclitaxel in the WSG-ADAPT HER2+/HR- phase II trial [23]. In this study, 134 patients with hormone receptor-negative HER2-positive early breast cancer were randomized to receive neoadjuvant dual antibody therapy, with or without chemotherapy. Pathologic complete remission was measured after 12 weeks. For patients treated with dual antibody blockade alone, chemotherapy was added to neoadjuvant therapy if pathologic complete remission was not achieved. Patients who achieved pathologic complete remission after antibody therapy alone had survival rates similar to those reported for patients in the chemotherapy arm.

However, many patients received postoperative chemotherapy with epirubicin and cyclophosphamide (with additional paclitaxel in the de-escalated neoadjuvant treatment arm) without clear evidence of which patients were benefiting from this anthracycline therapy.

## 7. Locoregional Therapy

In terms of locoregional treatment, de-escalation can be applied to surgery as well as radiotherapy [24]. Unfortunately, evidence from prospective trials regarding this topic is currently limited. For surgery of the primary breast tumor, it is considered standard of care to operate within the new borders of the tumor after neoadjuvant therapy. The EBCTCG meta-analysis of neoadjuvant vs. adjuvant chemotherapy showed increased rates of local relapse in patients who were initially planned for mastectomy and later received breast-conserving surgery [24]. This was mostly interpreted as a result of outdated postoperative imaging and margin assessment, as well as the omission of surgery in patients with a clinical complete response in some of the trials.

On the other hand, in a multicenter prospective study, Heil et al. were able to show that 37 of 208 residual tumors were not detected using image-guided vacuum biopsy in patients with a clinical complete response [25]. Procedure and patient selection seem to be essential to confidently evaluate a pathologic complete remission.

After completion of systemic neoadjuvant therapy, the tumor can be staged with imaging and pathology. MR imaging represents the diagnostic standard in evaluating tumor response. However, overestimation and underestimation of residual tumor size is common. Imaging techniques that combine functional aspects or incorporate artificial intelligence systems are desirable for better diagnostics [26].

The de-escalation of axillary surgery is applied for patients with clinically involved lymph nodes that develop a clinical complete response in the axilla [27]. While axillary lymph node dissection was performed historically, this has been replaced by sentinel lymph node biopsy, mostly with the additional removal of initially involved lymph nodes (targeted axillary dissection). Axillary lymph node dissection is still routinely performed in patients with positive lymph nodes after neoadjuvant chemotherapy.

However, emerging data suggest that if initially affected lymph nodes are negative subsequent to targeted axillary dissection after neoadjuvant chemotherapy, axillary dissection may not be necessary. Nevertheless, a randomized trial by the ALLIANCE group (NCT01901094) analyzing whether radiotherapy to the axilla may provide similar efficacy as dissection with a reduction in treatment-related morbidity is currently enrolling patients. The omission of breast surgery has been discussed in specific clinical scenarios [25]; however, results from carefully conducted clinical trials are pending, with one obstacle being the prediction of pathologic complete response with imaging and biopsy techniques [28].

For radiotherapy, treatment de-escalation may result in the omission of regional nodal irradiation after breast-conserving surgery or of post-mastectomy radiotherapy [29]. For patients with locally advanced breast cancer, withholding post-mastectomy radiotherapy results in inferior outcome, as shown in several retrospective analyses [30]. However, the benefit of regional nodal irradiation after breast-conserving surgery or of post-mastectomy radiotherapy in patients with cT1-2 cN1 breast cancer that are ypN0 or have a pathologic complete response remains unclear [30]. The NSABP B-51/RTOG 1304-trial (NCT01872975) addresses this specific question.

Similarly, neoadjuvant therapy poses organizational and logistical challenges for the clinical centers to optimize the timing between the different disciplines. Evidence suggests that surgery within eight weeks after initiation of systemic therapy is advantageous over later surgery [31].

## 8. Future Approaches

Unfortunately, our therapeutic options, and thus the range of post-neoadjuvant therapies, are still limited, but new therapeutic approaches such as immunotherapeutic treatments (e.g., tumor-specific vaccinations) are being tested in the post-neoadjuvant setting in these high-risk situations. In these circumstances, in contrast to metastatic disease, the systemic tumor cell count is rather low, and the effectiveness of these immunotherapeutic approaches is probably greater. On the other hand, these perspectives also imply the dynamic adjustment and modification of therapies, which may concern patients. The close involvement of the patient in the medical decision-making process, especially when the treatment regimen remains unclear at the beginning of the therapy and is independent from iterative treatment response rates, is a new challenge that we will face more often within our future clinical routine. Despite the tremendous euphoria associated with the increasing implementation of post-neoadjuvant strategies, long-term experiences are very limited.

It has recently been recognized that the binary approach of pCR and non-pCR does not stratify the risk groups optimally. Yau et al. introduced a categorization according to the residual disease burden, which categorizes the tumor into three grades, according to the size of the residual tumor [32]. The authors recently found that with an increase in the residual cancer burden, the outcome worsened.

Circulating tumor DNA (ctDNA) and circulating tumor cells are emerging methods to determine this. In a study of 196 patients, Radovich et al. showed that patients with no evidence of ctDNA at the time of surgery had significantly better distant free (*p* = 0.009) and overall survival (*p* = 0.002) [33]. By determining the ctDNA, the findings were even further refined.

Furthermore, while the correlation between the presence of residual tumor and prognosis seems distinct in triple-negative breast cancer, it does not appear clear in hormone receptor-positive HER2-negative tumors. For these patients, Marme et al. demonstrated better prognostic predictive validity for a scoring system consisting of clinical stage, pathology after treatment, hormone receptor status and grading [34]. In this way, resistance mechanisms could be identified, which then enable more effective post-neoadjuvant treatments. The first two large, randomized studies that tested this individualized, post-neoadjuvant therapeutic approach were the KATHERINE [5] and the PENELOPE-B studies [7]. Both studies attempted to improve the prognosis of these patients in the persistent high-risk situation after neoadjuvant standard therapy by targeted modification of the adjuvant or post-neoadjuvant treatment.

As it must be generally recognized that, as a consequence of a long neoadjuvant therapy, the risk is higher that patients discontinue due to complications or missing compliance, and therefore subsequently do not receive local therapy, close monitoring of patients becomes even more important. Furthermore, it remains to be clarified whether the promising results are transferable to routine clinical practice. While controversial results were published concerning the benefit of intensified adjuvant therapies in the absence of complete remission, generally worse tolerability became apparent. Therefore, it will continue to be a challenge during clinical routines to balance the unclear benefit against the higher risk of complications. The high toxicity associated with the escalation of therapy in the CREATE-X study led to a high discontinuation rate of 18% after six cycles and 25% after eight cycles of capecitabine. However, with today’s protocols, the tolerability appears to have been improved.

These results are consistent with the recently published experience concerning the intensification of adjuvant treatment with palbociclib in patients with residual disease after neoadjuvant chemotherapy. While oncologic control rates remained comparable, there was significantly higher toxicity attributable to palbociclib.

In a meta-analysis including 4756 women from ten randomized trials, neoadjuvant chemotherapy appeared to be associated with a higher risk of local recurrence than adjuvant chemotherapy (*p* = 0.0001). The results can be explained mainly by the lack of surgery after complete remission in some of the included studies. Furthermore, the conclusions of this meta-analysis have to be considered with great caution, as the included studies mostly contain historical chemotherapy protocols and hardly any targeted treatments. Nevertheless, these are exciting times, as the desire for the individualization of treatments, i.e., therapy escalation with high individual risk or therapy resistance as well as de-escalation in the case of low risk or therapy response, becomes a reality; maybe in small, but visible, steps, at the moment.

Currently, several promising post-neoadjuvant trials are ongoing. The SASCIA trial is an encouraging study for patients with HER2-negative breast cancer and residual tumor after neoadjuvant therapy. These patients will be randomized to determine whether adjuvant therapy with sacituzumab govitecan, which demonstrated efficacy in the metastatic setting, is superior to investigator choice.

Patients with HER2-positive breast cancer and non-pathologic complete remission have been randomized to adjuvant treatment with trastuzumab deruxtecan or trastuzumab emtansine after neoadjuvant chemotherapy and local therapy in the Destiny Breast 05 study since December 2020.

However, in the future, we will have to analyze both the initial disease and the residual disease after surgery more precisely at the molecular level and also with regard to the tumor microenvironment. This is where modern, high-throughput processes developed by basic research help us to detect heterogeneous tumors. These are, for example, site-specific analyses of the tumor microenvironment, individual tumor cell analyses and screening for individual tumor cells or tumor-specific molecules in the blood. It is increasingly recognized that differentiation between pathological complete remission or residual tumor is potentially insufficient.

## 9. Conclusions

There is still a long way to go before we can realize a truly individualized therapy using the most appropriate therapy at optimal time and sequence for all our patients. In view of the explosive growth in knowledge in oncology, only an interdisciplinary network can bundle all forces for the benefit of our patients with breast cancer. In regard to this special issue, we would like to discuss the current situation and approaches.

## Figures and Tables

**Table 1:** Important trials of post-neoadjuvant therapy (Only phase III trials are listed).

Trial	N	Cohort	Design	Result
CREATE-X Trial Masuda et al. 2017 [6]	910	HER2-neg. BC, residual invasive disease after neoadjuvant therapy	Capecitabine 1250 mg/m^2^ b.i.d. d1-14 for 6-8 cycles vs. control	DFS HR 0.70 (0.53–0.92) OS HR 0.59 (0.39–0.90)
	286	Subgroup with TNBC		DFS HR 0.58 (0.39–0.87) OS HR 0.52 (0.30–0.90)
EXTENET Chan et al., 2020 [8]	2840	HER2-pos. BC + RD	Neratinib vs. placebo	DFS advantage
NaTaN study (GBG 36/ABCSG 29) Von Minckwitz et al., 2016 [9]	693	RD	Zolendronic acid vs. observation	No difference
KATHERINE Study von Minckwitz 2019 [5]	1486	HER2-pos. BC, residual invasive disease after neoadjuvant therapy	Trastuzumab emtansine (T-DM1) vs. trastuzumab for 14 cycles	iDFS HR 0.50 (0.39–0.64) More grade 3–4 toxicity with T-DM1
ECOG-ACRIN EA1131 Mayer et al., 2021 [10]	410	Clinical stage II/III TNBC with ≥1 cm residual disease in the breast	Carboplatin or cisplatin every 3 weeks for 4 cycles vs. capecitabine 1000 mg/m^2^ b.i.d. d1-14 for 6 cycles	iDFS HR 1.06 (0.62–1.81) More grade 3–4 toxicity with platinum
PENELOPE-B Loibl et al., 2021 [11]	1250	HR-pos. HER2-neg. BC with residual disease; CPS-EG score of 3 or of 2 with ypN+	Palbociclib 125 mg d1-d21 for 13 cycles vs. placebo	DFS HR 0.93 (0.74–1.17) More grade 3–4 toxicity with Palbociclib
OlympiA Tutt et al., 2021 [4]	1836	HER2-negative with *BRCA1* or *BRCA2* after local treatment and neoadjuvant or adjuvant chemotherapy.	Olaparib vs. placebo.	IDFS HR 0.41 to 0.82 *p* < 0.001 ) DFS (0.39 to 0.83; *p* < 0.001)) Death (HR CI, 0.44 to 1.05 *p* < 0.001) No substiantial increase in adverse events
Keynote 522	1174	Triple-negative	Neoadjuvant: chemotherapy+placeb adjuvant: placebo vs. neoadjuvant: chemotherapy + pembrolizumab adjuvant: prembolizumab	OS (HR 0.72 [95% CI, 0.51–1.02) Grade ≥3 treatment-related AE Pembrolizumab: 77.1% Placebo: 73.3%
IMpassion 031 Mittendorf et al., 2020 [12]	455	Triple-negative	Neoadjuvant chemotherapy plus intravenous atezolizumab chemotherapy plus	Pathologic complete response-rate superior for chemotherapy plus atezolizumab (*p* = 0.0044)
A-Brave Trial Conte et al., 2020 Not published	474	Triple-negative after surgery and adjuvant TNBC neoadjuvant chemotherapy + surgery no PCR	Avelumab vs. observation	Results pending

**Table 2:** Ongoing trials.

SASCIA NCT04595565	HER2 neg. following neoadjuvant chemotherapy and local therapy	Arm A: Sacituzumab govitecan (days 1, 8 q3w for eight cycles); Arm B: treatment of physician’s choice
DESTINY-Breast05 NCT04622319	HER2-+ without complete response after neoadjuvant therapy	Arm A trastuzumab deruxtecan Arm B: trastuzumab emtansine

## Data Availability

All data and materials can be accessed via C.M. and E.B.
